# 860. Molecular Characterization of Isolated *Staphylococcus aureus* Specimens Using Matrix-Assisted Laser Desorption/Ionization Time-of-Flight Mass Spectrometry (MALDI-TOF MS)

**DOI:** 10.1093/ofid/ofad500.905

**Published:** 2023-11-27

**Authors:** Amber Dawning, Xiting Lin, Robert C Jerris, Kevin Thornton, Noor Mohamed, Lilly Immergluck

**Affiliations:** Morehouse School of Medicine, Atlanta, Georgia; Morehouse School of Medicine, Atlanta, Georgia; Emory University School of Medicine/Children's Healthcare of Atlanta, Atlanta, Georgia; University of Washington, Seattle, Washington; Morehouse School of Medicine, Atlanta, Georgia; Morehouse School of Medicine, Atlanta, Georgia

## Abstract

**Background:**

*Staphylococcus aureus* (*S. aureus*) is a common cause of skin and soft tissue infections (SSTIs). Both the β-lactam resistant form, methicillin resistant *S. aureus* (MRSA), and non-resistant form, methicillin susceptible *S. aureus* (MSSA) are associated with disparate infections across age and race; community-associated infections are often due to *S. aureus* US300. Matrix-Assisted Laser Desorption/Ionization Time-of-Flight Mass Spectrometry (MALDI-TOF MS) is a used in clinical microbiology laboratories to identify bacterial species, but limited data is available on if this tool can identify different strains of *S. aureus*. *We hypothesize that MALDI-TOF MS can be used to identify strain-specific peaks of S. aureus USA300.*

**Methods:**

Children were enrolled from emergency departments and swabbed for *S. aureus* (anterior nares, axilla, perirectal, oropharyngeal, and wounds) during two time periods (2006-2008 and 2015-2016). MALDI-TOF MS was performed on *S. aureus* cultured from study participants. Default parameters used for variance stability, smoothing, baseline subtraction, and normalization in R 4.4.3. and MALDIquant v1.22 were applied to raw data. Peak detection and alignment were performed with a signal-to-noise ratio of three and tolerance of 300 ppm. Binning and machine learning algorithms were used to identify intensity differences of each peak identified; significance of peaks was determined using Chi Square statistical analyses, comparing MRSA and MSSA, and then between all *S. aureus* isolates compared to USA300. Statistical significance was considered with p-value < 0.05 using R and SAS 9.4.

**Results:**

From 57 MRSA and 53 MSSA isolates, 320 peaks were identified. 31 significant peaks were identified, with 14 unique peaks for MRSA and 17 for MSSA. With *S. aureus* USA300, eight of the 31 peaks remained statistically significant.

**Conclusion:**

MALDI-TOF MS can identify unique peak differences between MRSA and MSSA. This tool can be potentially used to distinguish *S. aureus* at the strain level in a clinical setting. MALDI-TOF MS can be an impactful and rapid diagnostic tool to detect antibiotic resistance in *S. aureus* in a clinical setting.

**Disclosures:**

**Lilly Immergluck, MD,MS**, Melinta: Grant/Research Support|Melinta: Grant/Research Support

